# Effects of an evidence-based medicine workshop on Japanese pharmacy students’ awareness regarding the importance of reading current clinical literature

**DOI:** 10.1186/s40780-015-0024-5

**Published:** 2015-08-25

**Authors:** Naoto Nakagawa, Yuriko Murai, Makiko Yoshida, Hiroyuki Suzuki, Nariyasu Mano

**Affiliations:** Department of Pharmaceutical Sciences, Tohoku University Hospital, Sendai, Miyagi Japan; Graduate School of Pharmaceutical Sciences, Tohoku University, Sendai, Miyagi Japan

**Keywords:** Evidence-based medicine, Pharmacy students, Workshop, Small group discussion, Kawakita Jiro method

## Abstract

**Background:**

Drug literature evaluation has been taught at pharmacy schools in the United States, allowing pharmacy students to learn how to read clinical literature critically. In advanced pharmacy practice experiences, preceptors often assign pharmacy students to journal clubs in which they repeatedly train how to read such literature. This enables them to understand the importance of reading clinical literature prior to graduation. The objective of this study was to create evidence-based medicine (EBM) workshop that would enhance Japanese pharmacy students’ awareness regarding the importance of reading up-to-date clinical literature.

**Methods:**

The EBM workshop were designed as a one-day workshop consisting of student presentations regarding their opinions about reading clinical literature, a lecture on methods for reading required literature critically, and small group discussions using the KJ (Kawakita Jiro) Method. To evaluate the effectiveness of the EBM workshop, students were administered questionnaire surveys both before and after the workshop. The students also took a 15-question test on EBM. Regarding the questionnaires, students were asked to respond to dichotomous items (yes/no) and to indicate on a 7-point Likert scale the extent to which they agreed with statements about clinical literature. Student responses to both the pre- and post-workshop questionnaires were then compared to evaluate the effectiveness of the EBM workshop.

**Results:**

A total of 37 students participated in the EBM workshop. Significant improvement was seen between the pre- and post-workshop questionnaires in responses regarding whether they thought that pharmacists should read clinical literature regularly (pre-workshop: 5.70 ± 0.17 versus 6.51 ± 0.13 post-workshop; *p* < 0.0001), whether they were confident in their ability to read clinical literature (1.81 ± 0.15 versus 3.92 ± 0.18; *p* < 0.0001), and whether they could discuss treatment with nurses and physicians based on the results of clinical literature if they were a hospital pharmacist (2.49 ± 0.22 versus 3.86 ± 0.21; *p* < 0.0001). Significant improvement was also seen in scores on the EBM tests (11.4 ± 0.29 versus 12.6 ± 0.22; *p* < 0.0001).

**Conclusion:**

Our EBM workshop significantly enhanced student awareness regarding the importance of reading up-to-date clinical literature. It is therefore expected that students who participated in our EBM workshop will contribute to improvements in the quality of the pharmacy profession in the future.

## Background

Since 2006, pharmaceutical education in Japan has undergone a number of reforms, and the duration of pharmaceutical education has changed from 4 to 6 years. This revised version of pharmaceutical education is based on Model Core Curriculum (MCC) [1]. This new curriculum was introduced to enable pharmacy students in Japan to obtain more clinical experience and knowledge.

Evidence-based medicine (EBM) is well known around the world. Drug literature evaluation courses have been taught as in-class activities at almost every college or school of pharmacy in the United States [2, 3], and journal clubs have been so popular that both pharmacists and pharmacy students alike are able to easily access and critically read clinical literature [4, 5]. On the other hand, although the MCC in Japan includes an EBM category, it has not provided guidance on how to read clinical literature critically, and thus pharmacy students in Japan do not seem to have critical reading skills in relation to clinical literature. Therefore, some activities are necessary to enhance pharmacy students’ skills and awareness regarding the importance of reading up-to-date clinical literature.

Bookstarver et al. reported that EBM courses improve student performance in advanced pharmacy practice experiences [6] in the United States. In this report, we chose to adopt a small class size (5 to 6 students) because a small group style is considered to be one of the most effective instruction methods. In addition, the KJ (Kawakita Jiro) Method, which is known as an effective problem-solving technique, has often been utilized in the decision-making process in Japan [7]. Briefly, the KJ method involves the following four essential procedures: 1) Label making (using brainstorming); 2) Label grouping; 3) Chart-making; and 4) Written or verbal explanations. This method can promote a deeper understanding of problems and promote exposure to new viewpoints from other students. We considered that an activity involving the KJ Method might help facilitate student understanding of pharmacy-related topics. Therefore, for the purpose of enhancing student awareness of the importance of reading clinical literature regularly, we developed an EBM workshop for students and evaluated its effectiveness using questionnaire surveys administered both before and after the EBM workshop.

## Methods

### Orientation

**Fig. 1:**
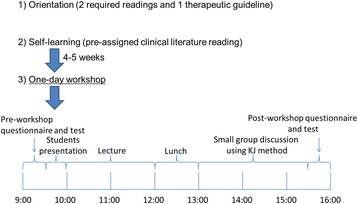
Role-play scenario activity in the EBM workshop

Before the study began, an orientation was held to inform pharmacy students of optional programs given at the beginning of pharmacy practice experiences. During the orientation, students were provided with an outline of the EBM workshop, which included a role-play scenario, required readings, and a suggested reading (Fig. 1).

**Fig. 2:**
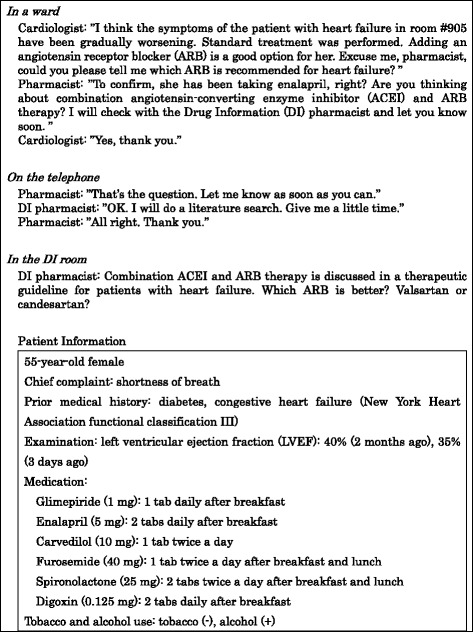
EBM workshop schedule

### Role-play scenario

Researching a physician’s drug information (DI) question is included in pharmacy practice experiences for 5th grade pharmacy students at Tohoku University Hospital; therefore, a role-play scenario involving a conversation between a physician, a pharmacist in a ward, and a DI pharmacist was created. Briefly, the patient being discussed was a 55-year-old female inpatient with congestive heart failure (New York Heart Association functional classification III) and diabetes. Her left ventricular ejection fraction was 40 % two months previously, and then, after developing shortness of breath, decreased to 35 % three days previously. The cardiologist considering her treatment was asking a question to the pharmacist in the ward (Fig. 2).

### Required and suggested readings

The pharmacy students were assigned two required readings related to the role-play regarding the clinical literature of valsartan [8] and candesartan [9]. A therapeutic guideline created by the Japanese Circulation Society for patients with chronic heart failure in Japan was also assigned as a suggested reading [10]. These materials were given to the pharmacy students during orientation before the EBM workshop. Based on these readings, the students had to make a decision during the role-play regarding which drug was better for the patient.

### EBM workshop

**Fig. 3:**
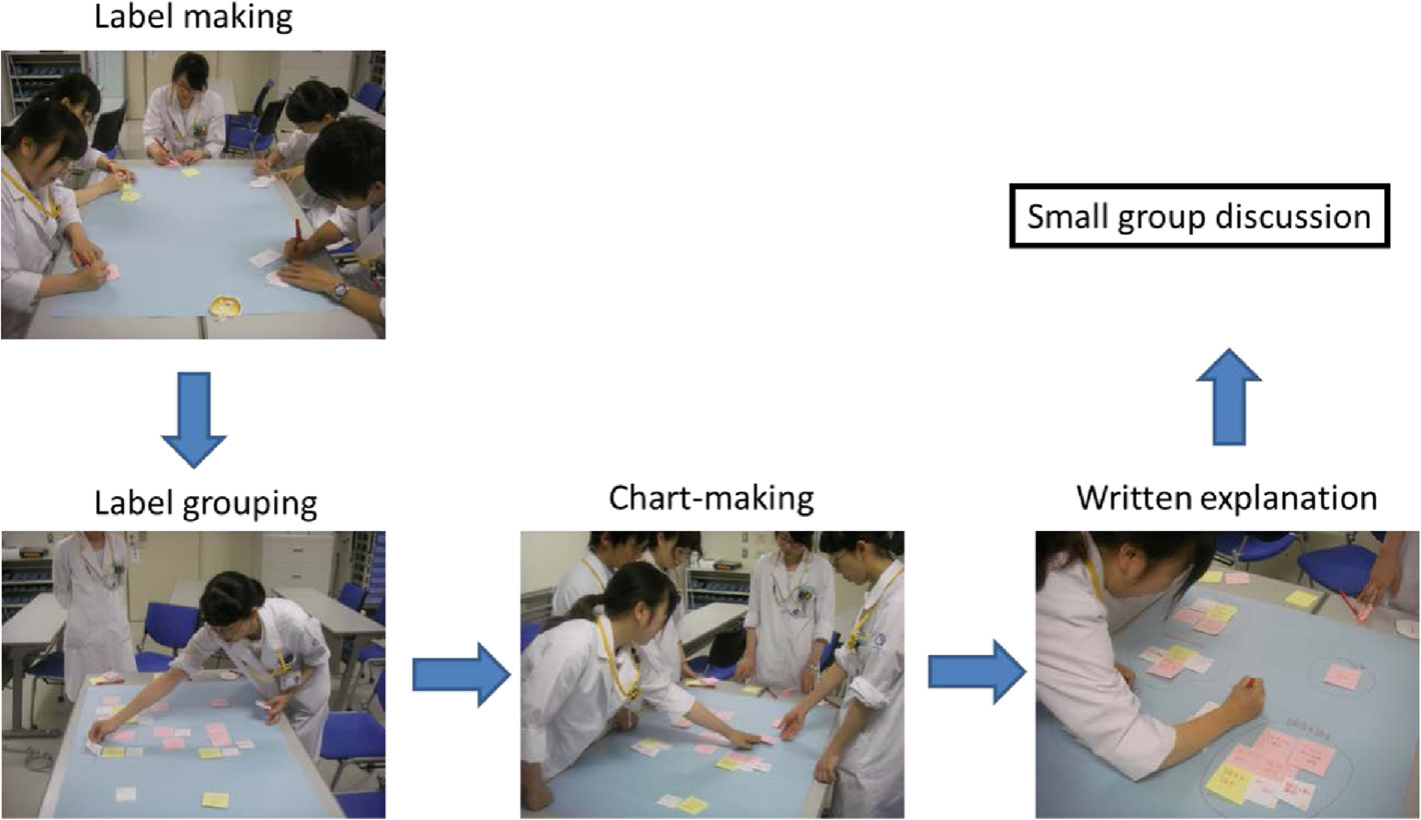
The four basic procedures of the KJ Method

The EBM workshop was part of a one-day workshop and one of the elective options in pharmacy practice experiences at Tohoku University Hospital. The EBM workshop consisted of two domains. The first domain involved student presentations regarding their opinions on critically reading clinical literature. Student presentations were based on required reading and a lecture by a preceptor was given to the students regarding how to read critically in a small group (5 to 6 students). The other domain involved using the KJ method (Fig. 3) to answer the following two questions: (1) “Is it possible to compare the clinical efficacy of valsartan and candesartan using package inserts and interview forms?”; and (2) “How can up-to-date evidence be obtained for questions arising or changes occurring after the therapeutic guidelines have been published?”

### Evaluation of the EBM workshop using-questionnaires

**Fig. 4:**
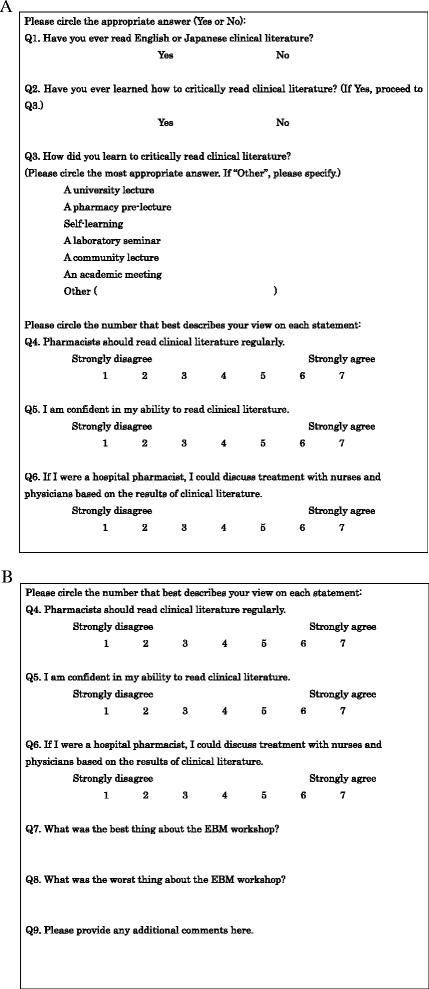
The questionnaires administered to pharmacy students. **a** Pre-workshop questionnaire; **b** Post-workshop questionnaire

To evaluate the effectiveness of the EBM workshop, we created questionnaires to administer both before and after participation. The pre-workshop questionnaire was designed to confirm the students’ background knowledge of and readiness to read clinical literature. The post-workshop questionnaire was almost as same as the pre-workshop questionnaire, but was designed to assess changes in student readiness. Both questionnaires asked students to indicate on a 7-point Likert scale (1: strongly disagree, 7: strongly agree) their level of agreement with statements regarding clinical literature (Figs. 4a and b).

### Pre-and post-workshop EBM test

**Fig. 5:**
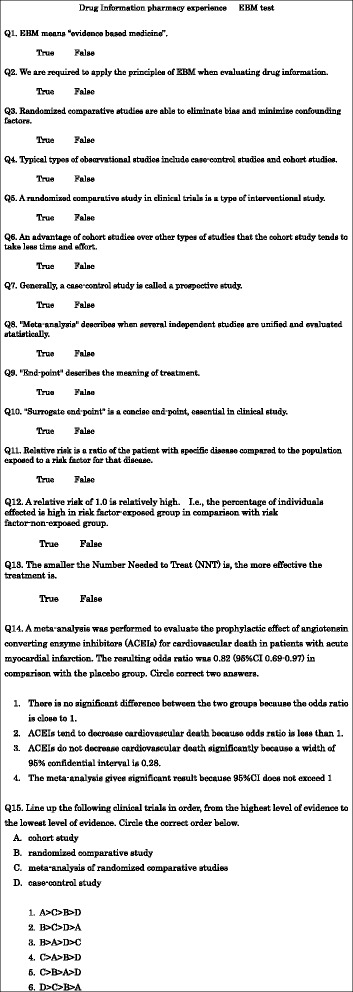
Pre- and post-workshop EBM test

Identical 15-question tests (Fig. 5) were administered to the students before and after the workshop to assess their baseline knowledge of EBM and to evaluate what they had learned, respectively.

### Evaluation of the EBM workshop in optional programs

An evaluation of the EBM workshop in terms of “Understanding”, “Satisfaction”, and “Necessity” was performed by pharmacy students in several optional programs at the end of pharmacy practice experiences. This evaluation used a 5-point Likert scale (1: Not understandable at all, Unsatisfying, Not necessary at all; 5: Completely understandable, Very satisfying, Extremely necessary).

### Statistics

The Wilcoxon signed-rank test was used to determine the statistically significant differences between the pre- and the post-workshop questionnaires, and the paired Student’s t test was used to determine the statistically significant differences between the pre- and post-tests. P values less than 0.05 were considered to indicate statistical significance.

### Ethical approval

This study was approved by the Tohoku University School of Medicine ethics committee (No.2013-1-050). The purpose of the study was explained to the pharmacy students during orientation for pharmacy practice experiences; participation in the questionnaire surveys was considered to indicate provision of informed consent.

## Results

A total of 37 pharmacy students participated in the EBM workshop, and 5 to 6 students per activity attended the small group discussions. The EBM workshop was held eight times between May 2013 and November 2013. Of 37 pharmacy students, 17 students participated in the EBM workshop in the first term of the long-term pharmacy practice experience and 20 students in the second term.

Partial results of the pre-workshop questionnaire, as well as a comparison of student responses between the pre- and post-workshop questionnaires, are shown in Table 1. We found that 57 % of the pharmacy students had previously read clinical literature, but 81 % had not learned how to read critically.

**Table 1:** Results of the pre- and post-workshop questionnaires on EBM (*n* = 37).

Questions	Yes (%)	No (%)	Pre-	Post-	*p* value
Q1. Have you ever read English or Japanese clinical literature?	21 (57 %)	16 (43 %)			
Q2. Have you ever learned how to critically read clinical literature?	7 (19 %)	30 (81 %)			
Q3. How did you learn to critically read clinical literature?	University lecture: 6				
	Laboratory seminar: 1				
Q4. Do you think that pharmacists should read clinical literature regularly?			5.70 ± 0.17	6.51 ± 0.13	<0.0001
Q5. Are you confident in your ability to read clinical literature?			1.81 ± 0.15	3.92 ± 0.18	<0.0001
Q6. If you were a hospital pharmacist, could you discuss treatment with nurses and physicians based on the results of clinical literature?			2.49 ± 0.22	3.86 ± 0.21	<0.0001
EBM test (15 questions)			11.4 ± 0.29	12.6 ± 0.22	<0.0001

EBM: Evidence-based medicine. Data are expressed as mean ± standard error (SE).

**Fig. 6:**
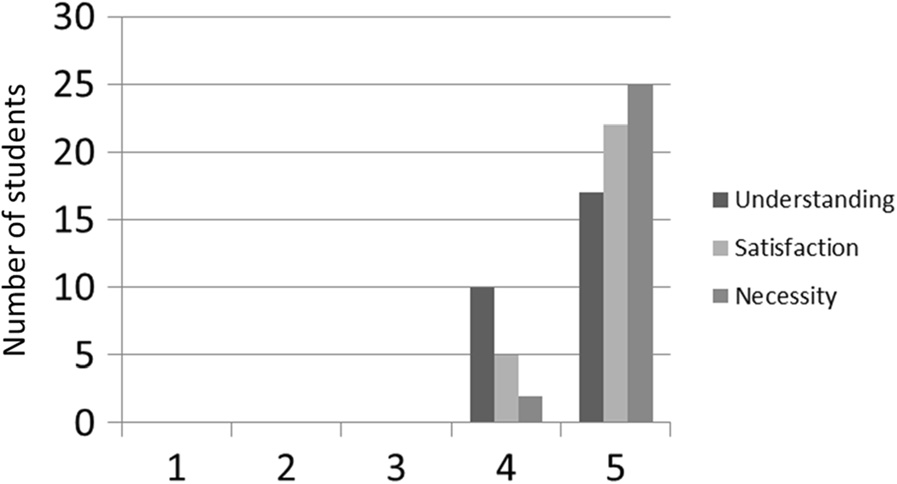
Student’s self-evaluations of the EBM workshop (*n* = 27) (1: Not understandable at all, Unsatisfying, Not necessary at all; 2: Difficult to understand, Somewhat unsatisfying, Somewhat unnecessary; 3: Neutral; 4: Mostly understandable, Satisfying, Somewhat necessary; 5: Completely understandable, Very satisfying, Extremely necessary)

The results of the EBM workshop evaluations by pharmacy students are shown in Fig. 6. Regarding “Understanding”, 10 students considered the EBM workshop to be mostly understandable, and 17 students considered it to be completely understandable. Regarding “Satisfaction”, 5 students considered the workshop to be satisfying, and 22 students considered it to be very satisfying. Regarding “Necessity”, 2 students considered the workshop to be somewhat necessary, and 25 considered it to be extremely necessary.

## Discussion

Although over 50 % of the pharmacy students had previously read clinical literature, a majority of the students had not learned how to read such literature critically, suggesting that most pharmacy students do not read clinical literature correctly. Therefore, the EBM workshop is one of the best opportunities to gain awareness of this inability, which suggests that new information from clinical literature would be applied to pharmacy practice when these students become pharmacists in the future. Based on the pre-workshop questionnaire, it was clear that most pharmacy students believe that pharmacists should read clinical literature regularly. Furthermore, student awareness of this point was significantly increased on the post-workshop questionnaire, which suggests the effectiveness of the EBM workshop. Therefore, it is expected that pharmacy students who participate in EBM workshop, such as those described in the present study, will read clinical literature critically and correctly and apply new information to pharmacy practice in the future.

In addition, although scores regarding student confidence in their ability to read clinical literature were low, they significantly increased from 1.81 ± 0.15 before the workshop to 3.92 ± 0.18 after the workshop; however, the post-workshop scores remained under 4, indicating an ongoing low level of student confidence. In addition, although scores regarding discussions with physicians and nurses were low, they significantly increased from 2.49 ± 0.22 before to 3.86 ± 0.21 after the workshop; however, similar to results for student confidence, these scores also remained low (under 4). These results suggest that our EBM workshop needs to be revised in order to significantly increase these scores. For example, one possibility would be to repeat EBM workshop twice or 3 times in pharmacy practice experiences. Repeated training in regard to clinical decision making based on clinical literature could also be of benefit.

Scores on the EBM pre-test were high (Table 1), indicating that Japanese pharmacy students already understood the concept of EBM. Scores on the EBM post-test were slightly higher, but significant, after the EBM workshop, suggesting that it was useful to summarize pharmacy students’ knowledge regarding EBM.

A majority of the pharmacy students considered the EBM workshop to be understandable, satisfying, and necessary (Fig. 6). In particular, “necessity” was the aspect most highly evaluated by the pharmacy students, suggesting that they were aware that reading clinical literature is necessary for pharmacists. In other words, if the revised MCC includes teaching how to read clinical literature critically, future students will understand the necessity of and appropriate methods for reading clinical literature correctly. Moreover, as these students begin their careers as pharmacists, the quality of health care can be expected to improve.

This study did have some limitations. First, as pharmacy students only had two required readings, comprehensive EBM items were not included in the EBM workshop. For example, relative risk and hazard ratios were used in the literature, so the preceptor had to provide descriptions of these ratios. Furthermore, although interpreting mean differences is also important for EBM, this was not covered in the workshop. Secondly, we did not evaluate how effective the KJ method is in the EBM workshop. Positive results might be due to the lecture by the preceptor or effectiveness of the KJ method or both. We will need to evaluate which domain is more effective in the next study. Finally, as the EBM workshop was conducted during one day, and the post-workshop questionnaire was administered immediately after its conclusion, the long-term effects of the workshop are unclear. A follow-up study might be necessary to determine any long-term effects.

## Conclusions

The results of this study suggest that our EBM workshop significantly enhanced student awareness of the importance of reading the clinical literature regularly. Therefore, reading up-to-date clinical literature prior to graduation is an important part of education for pharmacy students in Japan. It is therefore expected that pharmacy students who participated in our EBM workshop will contribute to improvements in the quality of the pharmacy profession in the future.
